# Optical projection tomography implemented for accessibility and low cost (*OPTImAL*)

**DOI:** 10.1098/rsta.2023.0101

**Published:** 2024-06-03

**Authors:** C. Darling, S. Kumar, Y. Alexandrov, J. de Faye, J. Almagro Santiago, A. Rýdlová, L. Bugeon, M. J. Dallman, A. J. Behrens, P. M. W French, J. McGinty

**Affiliations:** ^1^ Physics Department, Imperial College London, London SW7 2AZ, UK; ^2^ Francis Crick Institute, London NW1 1AT, UK; ^3^ Cancer Stem Cell Laboratory, Institute of Cancer Research, London SW7 3RP, UK; ^4^ Department of Life Sciences, Imperial College London, London SW7 2AZ, UK; ^5^ CRUK Convergence Science Centre & Division of Cancer, Department of Surgery and Cancer, Imperial College, London, UK

**Keywords:** open-source, microscopy, optical projection tomography, open hardware

## Abstract

Optical projection tomography (OPT) is a three-dimensional mesoscopic imaging modality that can use absorption or fluorescence contrast, and is widely applied to fixed and live samples in the mm–cm scale. For fluorescence OPT, we present OPT implemented for accessibility and low cost, an open-source research-grade implementation of modular OPT hardware and software that has been designed to be widely accessible by using low-cost components, including light-emitting diode (LED) excitation and cooled complementary metal-oxide-semiconductor (CMOS) cameras. Both the hardware and software are modular and flexible in their implementation, enabling rapid switching between sample size scales and supporting compressive sensing to reconstruct images from undersampled sparse OPT data, e.g. to facilitate rapid imaging with low photobleaching/phototoxicity. We also explore a simple implementation of focal scanning OPT to achieve higher resolution, which entails the use of a fan-beam geometry reconstruction method to account for variation in magnification.

This article is part of the Theo Murphy meeting issue 'Open, reproducible hardware for microscopy'.

## 1. Introduction

Volumetric optical imaging of approximately transparent fixed and live samples is becoming increasingly widespread in the life sciences, enabling biological phenomena to be studied in context, e.g. in disease models based on three-dimensional cell cultures [1], in small organisms [2] or in chemically cleared *ex vivo* tissues [3], which can be labelled with a wide range of stains and/or fluorophores. For three-dimensional imaging of ‘mesoscopic’ (approx. 1–10 mm scale) samples, a variety of three-dimensional fluorescence imaging techniques have been developed, including optical projection tomography (OPT) [4–6], scanning laser optical tomography [7] and light sheet microscopy (LSM) [8]. These diffraction-limited techniques can deliver three-dimensional images of transparent mesoscopic samples at higher imaging rates and with reduced photobleaching/phototoxicity compared to laser scanning microscopy. They have been applied to chemically cleared fixed samples and to live, semitransparent, organisms such as *Drosophila melanogaster* [9], *Caenorhabditis elegans* [10,11] and early-stage zebrafish (*Danio rerio*) embryos [12,13].

OPT is the optical analogue of X-ray computed tomography and entails the acquisition of projection images (wide-field two-dimensional transmission and/or fluorescence images in the case of OPT) of a sample from many viewing angles. The low phototoxicity associated with wide-field imaging makes it suitable for extended *in vivo* imaging studies, e.g. of zebrafish embryos [13] and adult zebrafish [14,15]; the low-cost and optomechanical simplicity makes it potentially accessible for lower-resourced settings. To this end, we present an open-source OPT implemented for accessibility and low cost (*OPTImAL*), including openly shared parts lists, CAD files and open-source software for image acquisition and analysis. *OPTImAL* complements other open-source resources for OPT (e.g. OptiJ [16]) and LSM (e.g. openSPIM [17]). In particular, *OPTImAL* uses low-cost light-emitting diode (LED) excitation sources and newly available low-cost cooled complementary metal-oxide semiconductor (CMOS) cameras. The image data acquisition is controlled by a plug-in for MicroManager [18], a widely used open-source microscopy platform. The instrument provides imaging arms with ×4 and unity magnification, enabling imaging of samples from less than 1 mm to greater than 1 cm diameter. Our image data reconstruction software is written in an interactive plugin for MATLAB and includes the option to use compressive sensing, where OPT data acquisition can be accelerated by acquiring significantly less image data and using an iterative algorithm for three-dimensional reconstruction. We also report a simple hardware extension to increase the spatial resolution using focal scanning and provide fan-beam reconstruction software to address the lack of telecentricity in this extended configuration.

**Figure 1.**
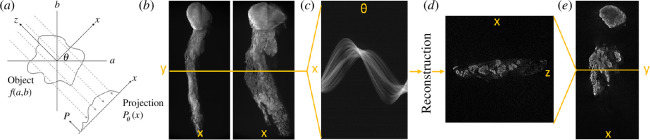
Principles of OPT imaging: projection views (*a*) of a sample mounted on a rotation stage are recorded forming (*b*) a set of projection images over 360° (projections at 0° and 55° shown here); (*c*) shows a single pixel height *x*–*θ* dataset extracted from this set of projections—referred to as a sinogram—from which an *x*–*z* slice of the original object can be reconstructed (d); stacking such *x–z* slices reconstructs the full volumetric image, from which a single *x*–*y* plane (of constant *z*) is shown in (*e*). Sample shown is epithelial cells labelled in a MMTV-PyMT mouse mammary gland.

In practice, OPT entails recording a series of projection images through a sample as it is rotated, then reconstructing the three-dimensional volume from these projection images using an algorithm based on the inverse Radon transform [19]. While the individual single projection images contain no explicit depth information, the complete set of projections contains spatial information from throughout the sample from which a volumetric image (with approximately isotropic resolution [20]) can be reconstructed. When the projection image data are filtered to optimize the weighting of the spatial frequency content, the technique is described as filtered back projection (FBP). This mathematical reconstruction formalism is for non-diffracting illumination, which is appropriate for telecentric imaging systems with extended depth of field (DOF). This has the advantage that a two-dimensional slice through the sample perpendicular to the axis of rotation can be reconstructed independently, which reduces the computational requirements as only a subset of the image projection data needs to be processed at a time. To use FBP to reconstruct OPT image data, the sample radius should be less than the DOF of the imaging optical system such that the rays can be assumed to be straight and parallel through the sample. This imposes a trade-off between achievable resolution and sample size for OPT. For all computed tomography techniques, there is also a requirement for a minimum number of projection images—that is determined by the angular resolution of the detectors—to reconstruct the volumetric image without loss of information. The principles of the technique are demonstrated in figure 1.

## 2. Basic *OPTImAL* implementation

Since the first publication of OPT [4] in 2002, there have been few commercially available OPT instruments. The deployment of OPT was partly held back by the long time required for computational reconstruction, typically hours per volumetric image. We started constructing OPT instruments in 2008 when we combined OPT with fluorescence lifetime imaging (FLIM) [21] and we used a GPU-based reconstruction algorithm in MATLAB that required only minutes to reconstruct an OPT volumetric image. This made our OPT instruments practical to use, with rapid image data acquisition and reconstruction. We progressed to image live zebrafish embryos with OPT and FLIM OPT [13] in a simple implementation where the rotating sample was mounted on a conventional inverted fluorescence microscope. We shared an open-source version of this instrument that used MicroManager for image data acquisition [22]. However, this specific design was limited to samples of less than 1 mm diameter and required the availability of an inverted fluorescence microscope. Here, we introduce *OPTImAL*, a more flexible, modular OPT platform with open hardware and open-source software that is designed to facilitate the convenient imaging of samples at multiple magnification scales in fluorescence (emission) and transmission (absorption) OPT modes. Our intention is to share the expertise gained using *OPTImAL;* we have developed and applied multiple OPT configurations to live zebrafish (larvae to adult) in agarose or water, or to fixed, cleared and labelled tissue, for which we have used a range of protocols for sample preparation, including BABB [4] and FLASH [23].

**Figure 2.**
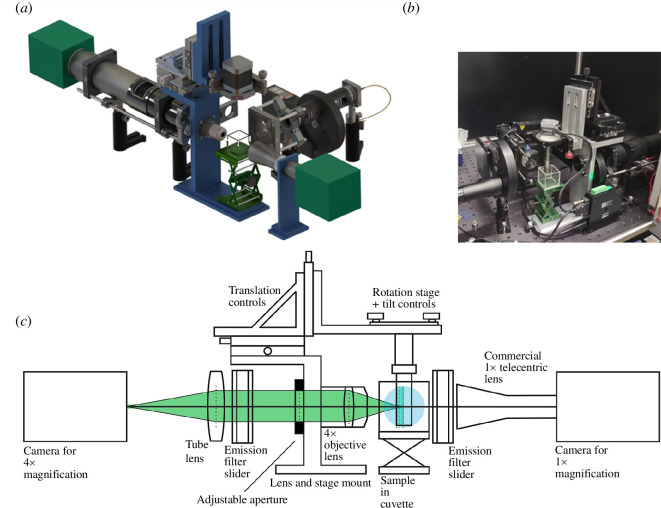
A rendering of CAD (*a*), photograph (*b*) and side view drawing (*c*) of the *OPTImAL* instrument; OPT requires widefield illumination, which can be provided by a range of light sources, with different delivery methods, depending on user needs and budget (see electronic supplementary material for exemplar illumination diagrams).

As illustrated in figure 2, *OPTImAL* is based on a central sample holder suspended from a motorized rotation stage with two telecentric imaging arms of different magnification: one comprising a microscope objective and tube lens for higher magnification; the other using a commercially available telecentric lens for lower magnification. There are filter sliders in each imaging arm and C-mounts for camera mounting. These two imaging arms may be used separately by switching a camera and filter set between the two arms, or simultaneously for imaging at different and complementary magnification scales using multiple cameras and filter sets at increased system cost.

Widefield fluorescence excitation light is provided by either a multimode diode laser or an LED source, which may be implemented in a variety of configurations, as is explored in the following sections and in the electronic supplementary material.

**Figure 3.**
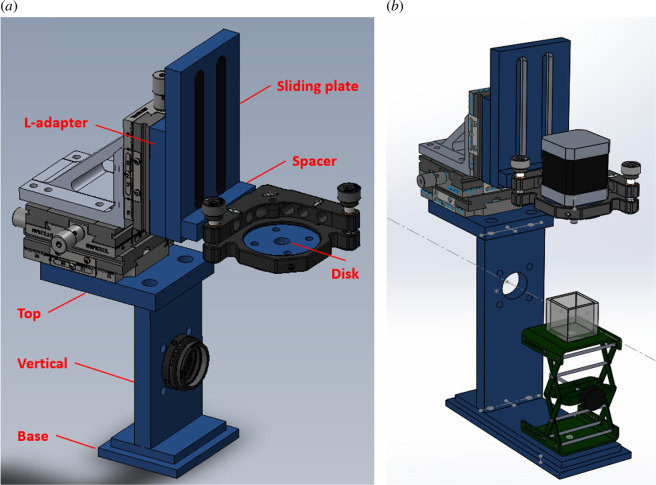
Three-dimensional renders of open-source custom rotation stage mount without (*a*) and with (*b*) cuvette for sample immersion, cuvette stand and example rotation stage. The individual parts to be machined are indicated in the left figure. Commercially available components (listed in the supplied parts list) are also rendered, to indicate how the complete mount is assembled.

The central mount for the rotation stage enables translation of the sample parallel and orthogonal to the optical axis, as well as tip–tilt adjustment. These adjustments are critical for the correct alignment of the sample axis of rotation, as outlined in the OPT imaging protocol (see electronic supplementary material). This mount comprises custom-machined aluminium components as well as commercially available parts (links to CAD files and component lists can be found in the electronic supplementary material). Figure 3 shows three-dimensional renders of this sample mount, indicating each custom-made part (shown in blue).

The optical components used for *OPTImAL* are all commercially available: for macroscopic (e.g. 1×) imaging, the commercial telecentric lens is mounted directly onto the imaging camera and incorporates an adjustable aperture that allows the DOF to be adjusted appropriately for the sample diameter. For microscopic (e.g. 4×) imaging, commercial microscope objective lenses are mounted directly onto the sample mount, and the tube lens is mounted on a (Thorlabs) rail system. Fluorescence emission filters, used to block any excitation light from reaching the camera, are mounted in a filter slider, which can be motorized for automated multispectral OPT image data acquisition. Projection images are recorded using an appropriate camera, which can be scientific grade (e.g. sCMOS sensor, Zyla 5.5 and Andor) or more recently available cooled CMOS cameras (e.g. CellCam Kikker and Cairn Research Ltd.). Electronic supplementary material, figure S1 shows different optical configurations of *OPTImAL* instruments.

**Figure 4.**
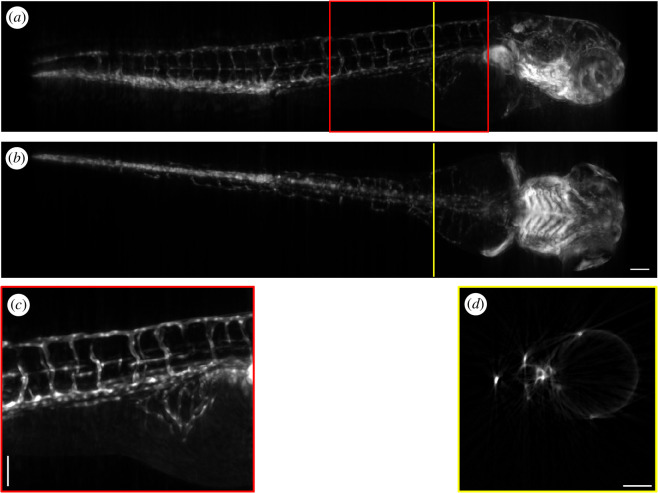
Volumetric image of labelled vasculature in an *in vivo* fli:GFP zebrafish embryo reconstructed from 400 projection images recorded at 4× magnification in under 3 min. Panels (*a,b*) show maximum intensity projections through the volumetric image, (*c*) shows an enlarged section, as indicated in red in the top panel and (*d*) shows a single reconstructed slice from the reconstructed volume, with the position in the full volume indicated in yellow in panels (*a,b*). Scale bar = 0.1 mm.

We first present the OPT instrument providing three-dimensional fluorescence imaging at 4× magnification. This system configuration uses a 4× objective lens (Nikon PlanFluor 4× 88–378) paired with a 200 mm focal length tube lens (Thorlabs TTL200), with excitation illumination at 465 nm provided by a multimode diode laser (from Lasertack GmbH, https://lasertack.com/en/standard-modules/) mounted in an optical fibre-coupled laser bank (Cairn Research Ltd., TriLine). The excitation light is delivered to the sample via a 0.8 mm diameter multimode optical fibre. The light emerging from the optical fibre is collimated using a 51 mm focal length Fresnel lens. To remove the laser speckle in the illumination, the multimode optical fibre was mechanically vibrated on a timescale faster than the camera exposure. Figure 4 shows volumetric images of the vasculature in a 3 days post fertilization (dpf) fli:GFP zebrafish embryo [24] acquired *in vivo*, imaged with this system configuration. The figure shows maximum intensity projections as well as a slice through the three-dimensional reconstructed volume. Electronic supplementary material, video S1 shows a rotating view. This OPT image data comprised 400 projection images acquired using an Andor Zyla 5.5 sCMOS camera, with an exposure time of 150 ms, and was reconstructed on a desktop PC (32 GB RAM, NVIDIA GTX 980 4 GB GPU) in under 3 min.

### (a) OPT with low-cost (cooled CMOS) camera

As many of the parts in our *OPTImAL* OPT system can be acquired or manufactured at low cost, the camera used to record the projection images is often a large portion of the total system cost (as can be seen in the component list provided). There is therefore motivation to use lower-cost cameras, rather than a scientific CMOS camera as was used to acquire the dataset presented in figure 4.

Typically, CCD and sCMOS cameras are used in microscopy owing to their low noise and high dynamic range characteristics, with CMOS sensors more typically used for video applications like machine vision where their higher noise—particularly thermal noise owing to lack of sensor cooling—is not a significant problem. Recently, however, lower-cost CMOS cameras with sensor cooling have become commercially available, such as the CellCam Kikker (Cairn Research Ltd), which (at less than £4000) costs significantly less than a scientific CCD or sCMOS camera. Originally developed to serve the astrophotography community, this new generation of cooled CMOS cameras has recently been adapted for fluorescence microscopy. The CellCam Kikker features a thermoelectrically cooled, back-illuminated SONY IMX492 CMOS sensor (90% quantum efficiency, 2.315 or 4.63 µm pixel size, 13 × 19 mm sensor size) and has previously been successfully deployed in a single-molecule localization microscope [25]. Here, we demonstrate its suitability for OPT imaging applications.

**Figure 5.**
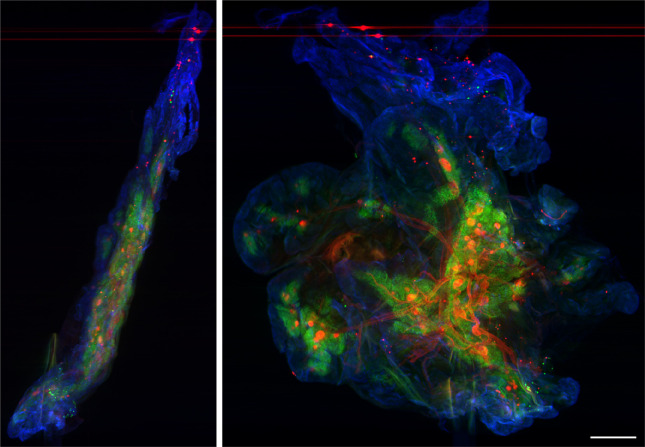
Two orthogonal views through volumetric fluorescence OPT image of fixed cleared mouse pancreas tissue. CK19 (blue), pancreatic amylase (green) and c-peptide (red) were labelled in the sample. The illumination was provided by the Cairn TriLine laser system, and projection images were recorded using the CellCam Kikker CMOS camera. Scale bar = 1 mm.

Figure 5 shows an OPT dataset imaged at 1× magnification using a commercial telecentric lens (Edmund Optics 58430 1.0 × SilverTL™ Telecentric Lens) attached directly to the CellCam Kikker. This exemplar three-colour fluorescence dataset was recorded using the CellCam Kikker, with multimode diode laser excitation at 488, 546 and 647 nm. The separate colour channels show CK19, pancreatic amylase and c-peptide (blue, green and red, antibody labelled with AF488 donkey anti-rat, AF546 donkey anti-goat and AF647 donkey anti-rabbit, respectively) in fixed, cleared mouse pancreas tissue prepared using the FLASH protocol [23]. The three-colour channel data, each comprising 400 projection images, were acquired sequentially. Electronic supplementary material, video S2 shows a rotating volumetric reconstruction.

The same sample was also imaged using the sCMOS camera to provide a comparison between the performance of the CellCam Kikker and Zyla sCMOS cameras, shown in electronic supplementary material, figure S3. Both cameras provided similar performance, producing satisfactory OPT images.

### (b) Fluorescence imaging with LED excitation light sources

As a wide-field imaging technique, OPT can take advantage of relatively low-cost excitation sources, including lamps and LED sources, neither of which require the stringent safety requirements which must be considered when using lasers (although higher-power LEDs can be hazardous, and a risk assessment should be carried out). Thus, LED-based instrumentation can potentially be more easily and widely implemented in a range of research environments. Furthermore, LED sources are inherently spatially incoherent and do not require dynamic de-speckling to increase illumination uniformity.

**Figure 6.**
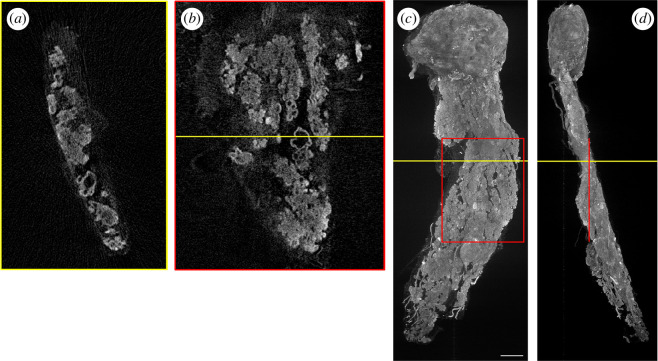
Volumetric OPT image of epithelial cells in MMTV-PyMT mouse mammary gland tissue. Illumination was provided by an LED source, and projection images were recorded on the CellCam Kikker CMOS camera. Panels (*a,b*) show two-dimensional planes from the three-dimensional dataset, positions in the volume indicated in (*c,d*), which show orthogonal projection views through the reconstructed three-dimensional dataset. Scale bar = 1 mm.

Therefore, to further address the potential of OPT as a low-cost technique for three-dimensional fluorescence imaging of biological samples, we used LED excitation in our OPT implementation using the CellCam Kikker cooled CMOS camera. Figure 6 shows a dataset recorded at 0.5× magnification using a telecentric lens (Edmund Optics 0.5× SilverTL), attached to the CellCam Kikker CMOS camera. LED illumination with a spectrum centred at 470 and 20 nm full width at half maximum (FWHM) bandwidth was provided by a surface-mount LED (Luminus SST-10-SB) mounted in a commercial package (Cairn Research Ltd, OptoLED). The radiation from the 1 mm^2^ active emission area (with a maximum output power of 530 mW) was coupled into a 1 mm core diameter, 0.39NA optical fibre (Lumencor 107263-4) by butting the fibre directly to the LED, realizing a measured coupling efficiency of approximately 5%. A short focal length singlet lens was used to collimate the light output from the optical fibre. To more efficiently deliver the LED output to a rectangular sample, a pair of cylindrical lenses was used to elongate the excitation beam along the long axis of the sample, although this is entirely optional. Figure 6 shows two-dimensional planes from, and maximum intensity projections through, a fluorescence volumetric image of a fixed, cleared and labelled sample of mammary epithelial cells in MMTV-PyMT mouse mammary gland tissue prepared using the FLASH protocol. Mammary cells were labelled with rat-raised keratin 8 antibody TROMA-I. This dataset indicates the performance of a lower-cost OPT instrument using both LED excitation and cooled CMOS cameras.

**Figure 7.**
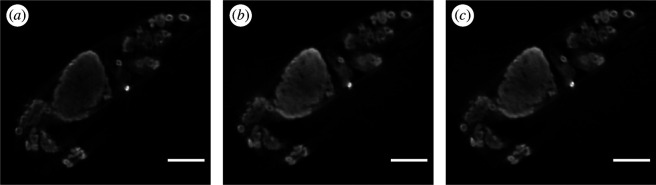
Comparison of the effect of different illumination sources on reconstructed slices of mammary epithelial cells in cleared MMTV-PyMT mouse mammary gland. Excitation illumination is provided by: (*a*) LED source (with 2 s camera integration time), (*b*) laser source (matched to LED power to achieve matched 2 s integration time) and (*c*) laser source (higher power, 400 ms camera integration). Scale bar = 0.5 mm [26].

We undertook a direct comparison of the OPT imaging performance when using laser and LED illumination. We found that LED excitation provided comparable imaging performance to laser illumination, although the camera integration time had to be increased because the LED illumination intensity was lower than the multimode diode laser source. Figure 7 shows slices from the volumetric data presented in figure 6, when illuminated (*a*) with the LED at a camera integration time of 2 s, (*b*) with the laser at a reduced intensity to approximately match the LED and (*c*) with the laser at full power and a reduced camera integration time of 400 ms. All datasets produced visually similar OPT data, but the image data acquisition time was reduced for the higher-power excitation, as camera integration time was reduced to acquire the same camera signal levels. In practice, photobleaching/phototoxicity considerations will guide the choice of excitation power levels used, noting that imaging live samples will require excitation power levels balancing the requirements to image fast enough to avoid motion artefacts while not compromising the sample through phototoxicity. When imaging fixed samples, LED illumination may provide a higher signal-to-noise ratio (through reduced photobleaching) but slower image data acquisition compared to using laser excitation at higher powers.

We note that while the LED source was fibre-coupled for light delivery to the sample in the results shown here, free-space LED solutions may also be used, and may be more appropriate for a given experiment. In the provided parts list, we outline a solution whereby filters and Fresnel lenses are mounted directly in front of the LED housing, allowing for convenient and flexible placement of the LED sources, as illustrated in electronic supplementary material, figure S2.

### (c) Open-source OPT software

While our open-source OPT hardware can be used with a range of software tools, we present here our own software implementation that has proved to be effective in controlling our OPT hardware and reconstructing OPT datasets. These software solutions also include support for a compressive sensing approach to reconstruct significantly undersampled OPT datasets, which enables faster volumetric imaging, e.g. of live samples. The shorter acquisition times realized through acquiring reduced numbers of projection images minimize sample movement, result in a lower light dose to live samples and reduce requirements to maintain live organisms under anaesthetic, which can improve sample recovery [27].

OPT projection image acquisition and hardware control are implemented through MicroManager [18]. This is a modular open-source software platform for the control of microscope hardware, for which there are existing device adapters for a wide range of imaging and optomechanical hardware. We provide an *OPTImAL MicroManager* OPT data acquisition plug-in that enables the user to align an OPT instrument and record projection images. This plug-in will work with any rotation stage and camera that can be controlled through MicroManager. We also provide an *OPTImAL sinogram checker* macro for *ImageJ* [28,29] (which runs in parallel with MicroManager) that enables the quality of recorded projection image data to be checked immediately after acquisition. Projection data can then be saved through MicroManager as a single OME TIFF file, or individual TIFF files for each projection image.

**Figure 8.**
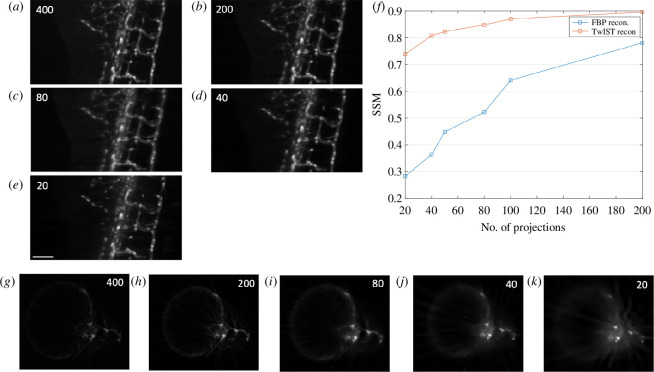
Demonstration of TwIST reconstruction for undersampled data. Maximum intensity projections through the reconstructed volume (*a–e*) and single reconstructed slices (*g–k*) of fli:GFP zebrafish embryo vasculature, reconstructed using TwIST algorithm from 200 (*b,h*), 80 (*c,i*), 40 (*d,j*) and 20 (*e,k*) projection images, down-sampled from a fully sampled set of 400 projections (shown reconstructed using FBP in a,g). Panel (*f*) shows the SSIM (structural similarity) index for these undersampled reconstructed slices, with reference to a 400 projection FBP reconstruction, when using both the TwIST and FBP algorithms. Numbers of projections are indicated in the corner of reconstructed images for clarity. Scale bar = 0.1 mm.

For volumetric OPT data reconstruction, we provide an interactive plug-in, *OPTtools*, that runs in MATLAB and saves volumes as OME TIFF, TIFF stacks or MATLAB files for subsequent visualization. Users are able to perform post-acquisition automatic or manual alignment (translation and rotation) of projection data, as well as image processing steps such as median filtering for the elimination of hot/stuck pixels, and pixel down-sampling to generate output volumes with a reduced number of voxels (i.e. reduced data size). Users are also able to correct for uneven illumination and offset if they upload background images. Reconstruction of volumetric images from OPT projection image data is done using FBP when sufficiently sampled, for which a range of filters are available. Sparse (undersampled) OPT projection image data can be reconstructed using TwIST [30], which is an iterative compressive sensing algorithm that we have previously demonstrated to be highly effective for this purpose [14,31]. Figure 8 shows an example of an OPT dataset of a fli:GFP zebrafish embryo from which images are reconstructed using 200, 80, 40 and 20 equally spaced projection images from the same dataset (of 400 projections, fully sampled). While the fully sampled image of figure 8*a,g* shows the fewest reconstruction artefacts, the figures retain most of the significant features even when reducing the number of projection images to 20 (20× down-sampling). Indeed, the structural similarity index measure (SSIM) index results indicate that reconstruction performance is comparable between 10× undersampling and reconstructing using the TwIST algorithm, and only 2× undersampling when using FBP. However, there is an associated reconstruction time penalty for using the TwIST algorithm. Using a desktop computer (featuring an NVIDIA GeForce GTX 980 GPU), reconstructing the 250 slices presented in figure 8 from 400 projection images using FBP took approximately 16 s. The TwIST algorithm run-time scales with the number of projections, and reconstruction times ranged from approximately 320 s for the same 250 slices from 20-projection images, to apprxoximately 11.5 min for a 100-projection image input, and approximately 23 min for a 200-projection image input. Both FBP and TwIST algorithms ran using GPU acceleration, which is implemented for optional use in the *OPTtools* plugin for CUDA GPUs.

The *OPTtools* MATLAB plug-in also features batch processing options for the reconstruction of multiple experiments, automatic segmentation of large projection images to allow reconstruction of volumes larger than available system RAM (particularly useful for reconstruction on more memory-constrained desktop computers) and automatic compensation of photobleaching during image acquisition for fluorescence OPT. Integration with Icy [32], an open-source image processing platform that provides rapid visualization and rendering of volumetric images, is also featured as part of the *OPTtools* plugin.

**Figure 9.**
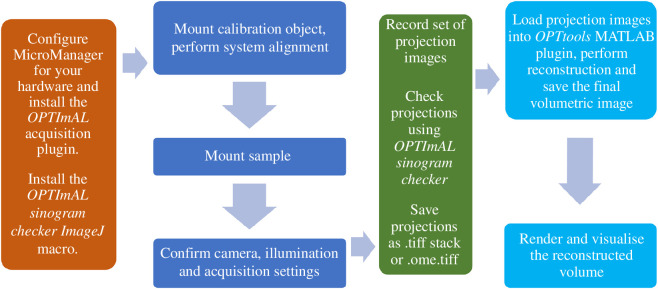
Basic workflow for *OPTImAL* implementation of OPT.

Figure 9 shows a schematic summarizing the workflow for *OPTImAL*, from initial instrument set-up to reconstruction and visualization of volumetric data. A link to a full protocol, as well as software manuals, is provided in the electronic supplementary material. The *OPTImAL* software tools are available at the *OPTImAL* GitHub repository [33].

## 3. Extending *OPTImAL* instruments with low-cost focal scanning

OPT requires projection image data that is in focus throughout the sample to correctly reconstruct the object using FBP. This is typically achieved by restricting the numerical aperture (NA) of the imaging optics to ensure that the DOF is at least equal to the radius of the sample. Typically, the DOF is set such that the closest half of a sample to the imaging optics is imaged in focus, and projection images are recorded through 360°. However, as NA also defines the diffraction-limited lateral resolution in a recorded image, the resolution of OPT images scales inversely with the sample radius. This constraint can be overcome by scanning a higher NA (lower DOF) focal plane through the object (or vice versa) since the OPT image reconstruction process inherently weights in favour of the higher-spatial frequency image data, with the out-of-focus light contributing to a low spatial frequency background that reduces the dynamic range of the image recording but does not significantly degrade the reconstructed images.

‘Focal scanning OPT’ has been previously demonstrated to provide higher-resolution OPT [8,34,35]. While the imaging focal plane can be scanned by translating either the objective lens or the sample itself, these mechanical approaches introduce significant cost and complexity to the set-up, particularly for large (cm) sample diameters. *OPTImAL* focal scanning is implemented by simply replacing the telecentric lens used in the lower-resolution imaging arm of figure 2 with a newly available telecentric lens (e.g. Edmund Optics 36192, MercuryTL™ 0.75×) that incorporates an electronically controlled tunable liquid lens. This simple adaptation of the basic *OPTImAL* configuration—at an incremental cost of less than £1000—builds on our earlier work implementing focal scanning OPT with a separate electrically tuneable liquid lens element in an image relay [36].

Axial translation of the imaging focal plane is achieved by varying the current applied across the liquid tuneable element, which changes its surface curvature and therefore its optical power (i.e. focal length). This principle is demonstrated in electronic supplementary material, figure S4. When recording projection images, the focal plane was swept across the entire depth of the sample by applying a triangle wave current signal to the liquid lens element. Ideally, this linear sweep results in equal time spent at each axial position during camera integration. The focal plane position was set to cycle many (approx. 20) times per camera integration to provide similar dwell times as a function of depth, even though the tuneable lens signal was not synchronized with the camera shutter. To minimize the contributions from out-of-focus light in the final OPT images, the projection images were deconvolved before tomographic reconstruction with a projection of the system three-dimensional point spread function (PSF) [37]. The PSF was modelled using known optical properties of the system, using the open-source MATLAB plugin PSF Generator [38].

### (a) Image telecentricity

**Figure 10.**
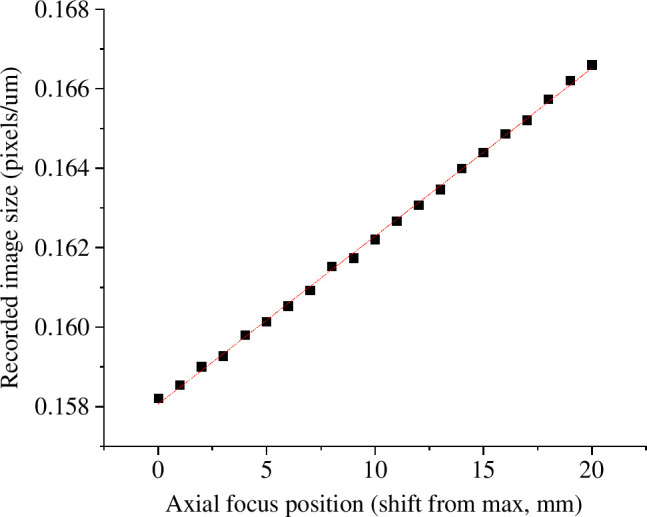
Measurement of recorded image magnification for a fixed-length object, as it is axially displaced, with the focal plane position changed to match the object position.

**Figure 11.**
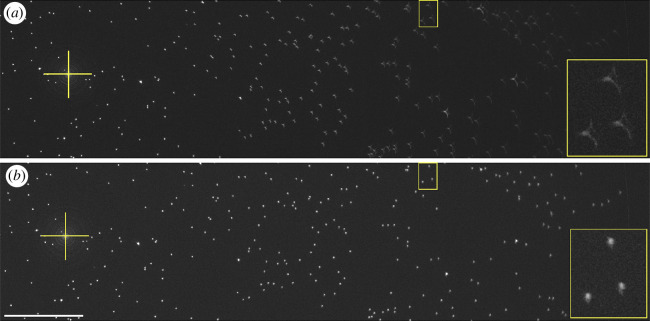
Maximum intensity projection through 500 slice stack from the centre of a 14 mm diameter phantom with 1 µm fluorescent beads. (*a*) Parallel projection assumed during reconstruction, resulting in artefacts away from the axis of rotation of the object; (*b*): fan beam projection geometry assumed during reconstruction (yellow cross indicates axis of rotation). Scale bar = 1 mm. Lower right inserts enlarge the beads shown in the smaller yellow boxes.

OPT systems are usually configured to be telecentric, such that the magnification is constant throughout the depth of the sample. When testing *OPTImAL* focal scanning, we observed that the image magnification varied slightly with axial focal plane position. This is quantified in figure 10, which shows a plot of the variation in the apparent size of a feature in the sample as it was axially displaced and repeatedly imaged with the focal plane position adjusted to keep the object in focus as a function of imaging depth. This change in magnification with the axial position of the imaging focal plane is a deviation from parallel projection geometry and produces artefacts in images reconstructed using the conventional FBP reconstruction, as can be seen in figure 11*a*.

The observation that the change in magnification is approximately linear with axial position (cf. figure 10) suggests that the projection geometry could be modelled as a cone beam, as is common in x-ray computed tomography. As cone-beam projections record information from multiple ‘heights’ in a sample onto each row of detectors (pixels), it is no longer possible to reconstruct individual two-dimensional slices from single camera pixel rows as is done with FBP. This is demonstrated in the reconstruction of sub-resolution fluorescent beads from across the FOV presented in electronic supplementary material, figure S5. While full three-dimensional reconstruction algorithms exist, e.g. FDK [39] implemented as part of the open-source ASTRA toolkit [40], the GPU memory required to store high-resolution optical tomography images during reconstruction is prohibitive without distributed computing and is not compatible with our goal of wide accessibility for *OPTImAL*. Fortunately, it is possible to approximate the projection geometry to a fan beam in the central plane through the FOV, supporting the two-dimensional reconstruction of a single slice in this regime (see projection geometry diagram in electronic supplementary material, figure S6). Figure 11*b* (and electronic supplementary material, figure S7) illustrates the successful reduction in reconstruction artefacts when reconstructing sub-resolution fluorescent beads from this central region of the FOV assuming a fan-beam projection geometry, with the origin of the fan beam empirically derived to minimize the observed reconstruction artefacts.

**Figure 12.**
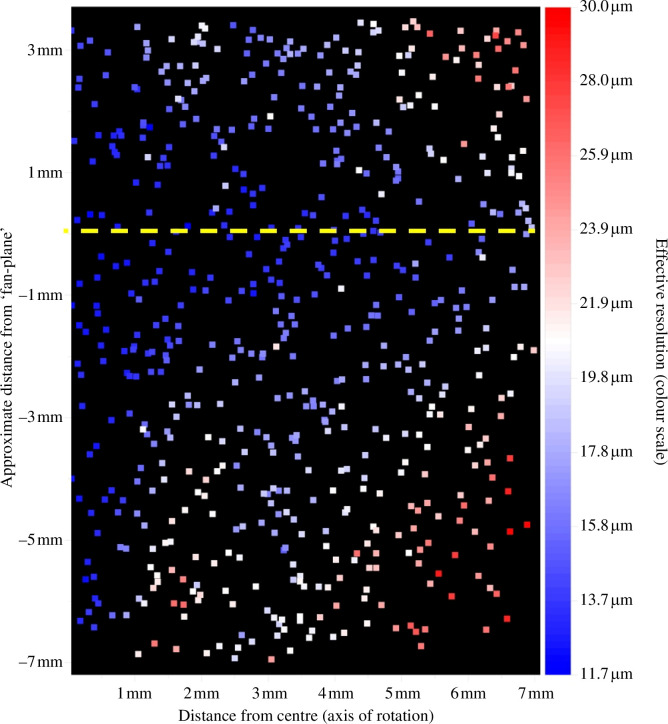
False colour plot of effective resolution of fan-beam geometry-based reconstruction across *OPTImAL*-focal scanning FOV represented by estimates of reconstructed bead FWHM of 1 µm diameter fluorescent beads. Yellow-dashed line corresponds to the central plane where the fan-beam geometry is valid.

Strictly, this approach will only be valid for the central two-dimensional slice in the plane of incidence of the imaging lens. In figure 12 we illustrate how the fan-beam reconstruction of figure 11*b* performs away from this central slice by plotting the diameter of the reconstructed images of sub-resolution (1 µm) fluorescent beads of the phantom imaged as a proxy for degraded imaging performance.

The reconstructed bead diameters were calculated from a maximum intensity projection through the reconstructed volume that was analysed using *PICASSO* [41,42], a tool developed for single-molecule localization microscopy. *PICASSO* detected each of the reconstructed beads and assigned a sigma value, from which an estimated FWHM of the bead image (which includes reconstruction artefacts) is calculated. In figure 12, the reconstructed bead images are colour-coded with the estimated FWHM, giving a visual representation of the variation in effective resolution across the reconstructed FOV. As expected, reconstruction artefacts are minimal close to the centre of the field of view, where the projection geometry is well described by a fan beam.

We note that the expected resolution of conventional OPT using a fixed focus telecentric lens of the same NA is 38 µm for a 7 mm DOF (set to equal the sample radius), while the *OPTImAL* focal scanning method presents a reconstructed volume with reconstructed bead FWHM below this limit across the entire field of view and approximately 12 µm for the slice at the central plane. If *OPTImAL* focal scanning is to be applied to samples of height greater than 2 mm, then the sample could be stepped along the direction of the axis of rotation and—for this set-up—imaged at 2 mm intervals to achieve maximum (approx. 12 µm) resolution throughout the sample volume. This could be achieved by manually adjusting the vertical translation stage on the *OPTImAL* set-up, or a motorized stage could be used for automated imaging at focal scanning resolution.

**Figure 13.**
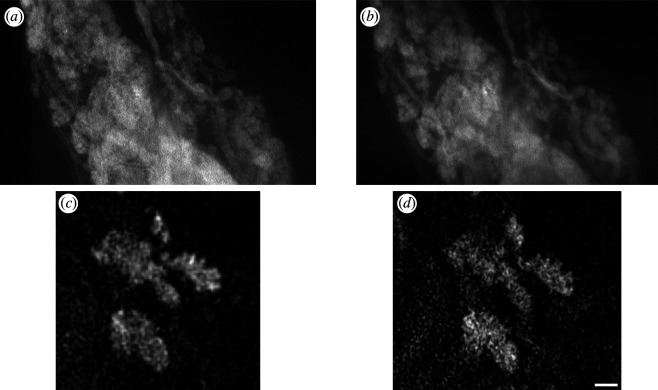
Comparison of conventional (fixed lens) (*a,c*) and focal scanning *OPTImAL* (*b,d*) when imaging mammary epithelial cells in a mammary gland from a MMTV-PyMT mouse. (*a,b*) compare maximum intensity projections through reconstructed volumes, while (*b,d*) compare single two-dimensional slices (scale bar 100 µm). All projection images were deconvolved prior to reconstruction.

This vertical displacement/focal scanning approach is demonstrated in figure 13, where mouse mammary gland tissue (previously shown in figure 6) was imaged using the focal scanning lens with the CellCam Kikker CMOS camera. Excitation illumination was provided by the fibre-coupled LED, with the camera integration time for each projection image set to 1 s; 640 projection images were recorded per 2 mm section, over 180° (as the projections contain in-focus information from throughout the sample). The approximately 13 mm long object was imaged in seven sections, vertically shifted by 2 mm using the manual translation stage incorporated into the *OPTImAL* instrument (only a section of the sample is shown in figure 13). Focal scanning *OPTImAL* measurements of sub-resolution fluorescence beads are presented in electronic supplementary material, figures S5 and S7 to provide further analysis of the effective resolution and reconstruction artefacts incurred for the different reconstruction geometries, and electronic supplementary material, figure S8 illustrates how artefacts present when reconstructing sample planes away from the central plane in a biological sample.

Because the fan-beam reconstruction is still at an early stage, we have not yet incorporated it in the *OPTtools* plug-in. However, the MATLAB code, which uses algorithms from the ASTRA toolbox [40], is available from the *OPTImAL* GitHub repository [33].

## 4. Conclusions

We have presented *OPTImAL*, a complete open-source OPT system for volumetric imaging of mm–cm scale samples that have been designed to be widely accessible using lower-cost research-grade components. Both hardware and software are modular and flexible in their implementation. The *OPTImAL* instrument, which was designed for fluorescence OPT, supports rapid switching between sample size scales, cameras and illumination sources, to enable the imaging of a wide range of samples. Our volumetric image reconstruction software incorporates post-processing capabilities and supports compressive sensing to reconstruct images from undersampled sparse OPT data.

We have demonstrated the potential to lower the cost of OPT instrumentation by using newly available cooled CMOS cameras that can replace much more expensive scientific-grade cameras and by using LEDs that can replace more expensive laser sources with minimal effect on performance beyond image data acquisition speed. For many samples, fluorescence imaging with lower excitation power for longer image acquisition times can be advantageous in terms of reduced photobleaching [43]. We have also presented a low-cost extension to higher-resolution focal scanning OPT through the use of a recently available focal scanning telecentric lens. We note that, while the system does provide higher-resolution volumetric reconstructions for samples beyond approximately 1.3 mm in radius, the lens is not quite telecentric while scanning, resulting in reconstruction artefacts that we have addressed with a modified reconstruction method based on fan-beam geometry. Future versions of the commercial focal scanning telecentric lens may mitigate these issues, or an alternative focal scanning solution can be assembled, e.g. [36], albeit at increased system cost and complexity. We note the potential to take advantage of machine learning in the reconstruction of OPT image data [44] and suggest that this may provide a route to more efficient reconstruction software, including for cone-beam reconstruction.

*OPTImAL* could be adapted for brightfield OPT, e.g. by adding a white light source such as an electroluminescent backlight, but we have not yet undertaken this. If fluorescence OPT is not required, it may be practical to use even lower-cost (uncooled CMOS) cameras, as we have used in other OPT instruments (unpublished).

Experiments involving zebrafish were conducted in accordance with UK Home Office requirements (Animals Scientific Procedures Act 1986, project licence P5D71E9B0). Laboratory mice were maintained according to home office regulations (PPL PP9490916 Behrens).

## Data Availability

Our software and code are available at [33]. Raw data (for reproducing figures) are available at [45]. Electronic supplementary material is available online [46].
